# Does cardiac surgery in newborn infants compromise blood cell reactivity to endotoxin?

**DOI:** 10.1186/cc3794

**Published:** 2005-08-09

**Authors:** Kathrin Schumacher, Stefanie Korr, Jaime F Vazquez-Jimenez, Götz von Bernuth, Jean Duchateau, Marie-Christine Seghaye

**Affiliations:** 1 Fellow in pediatrics, Department of Pediatric Cardiology, Aachen University, Aachen, Germany; 2 Fellow in internal medicine, Department of Pediatric Cardiology, Aachen University, Aachen, Germany; 3 Head of department, Department of Pediatric Cardiac Surgery, Aachen University, Aachen, Germany; 4 Former head of department, Department of Pediatric Cardiology, Aachen University, Aachen, Germany; 5 Director, Department of Immunology, University Hospital Brugmann and Saint-Pierre, Free University of Brussels, Brussels, Belgium; 6 Head of department, Department of Pediatric Cardiology, Aachen University, Aachen, Germany

## Abstract

**Introduction:**

Neonatal cardiac surgery is associated with a systemic inflammatory reaction that might compromise the reactivity of blood cells against an inflammatory stimulus. Our prospective study was aimed at testing this hypothesis.

**Methods:**

We investigated 17 newborn infants with transposition of the great arteries undergoing arterial switch operation. *Ex vivo *production of the pro-inflammatory cytokine tumor necrosis factor-α (TNF-α), of the regulator of the acute-phase response IL-6, and of the natural anti-inflammatory cytokine IL-10 were measured by enzyme-linked immunosorbent assay in the cell culture supernatant after whole blood stimulation by the endotoxin lipopolysaccharide before, 5 and 10 days after the operation. Results were analyzed with respect to postoperative morbidity.

**Results:**

The *ex vivo *production of TNF-α and IL-6 was significantly decreased (*P *< 0.001 and *P *< 0.002, respectively), whereas *ex vivo *production of IL-10 tended to be lower 5 days after the operation in comparison with preoperative values (*P *< 0.1). *Ex vivo *production of all cytokines reached preoperative values 10 days after cardiac surgery. Preoperative *ex vivo *production of IL-6 was inversely correlated with the postoperative oxygenation index 4 hours and 24 hours after the operation (*P *< 0.02). In contrast, postoperative *ex vivo *production of cytokines did not correlate with postoperative morbidity.

**Conclusion:**

Our results show that cardiac surgery in newborn infants is associated with a transient but significant decrease in the *ex vivo *production of the pro-inflammatory cytokines TNF-α and IL-6 together with a less pronounced decrease in IL-10 production. This might indicate a transient postoperative anti-inflammatory shift of the cytokine balance in this age group. Our results suggest that higher preoperative *ex vivo *production of IL-6 is associated with a higher risk for postoperative pulmonary dysfunction.

## Introduction

Cardiac surgery is associated with a systemic inflammatory reaction comprising activation of the complement system, stimulation of leukocytes, synthesis of cytokines, and increased interactions between leukocytes and endothelium [1,2]. In children, contact activation, ischemia/reperfusion injury and endotoxin released from the gut [3,4] are thought to be the major inductors of pro-inflammatory cytokines such as tumor necrosis factor-α (TNF-α) and IL-6 in the cardiac surgery setting. In newborn infants, morbidity after cardiac surgery is related to the importance of the intra-operative production of pro-inflammatory cytokines such as IL-6, as we have shown previously [5].

NF-κB is the main transcription factor of many inflammatory genes, such as that encoding TNF-α [6]. TNF-α induces secondary mediators of inflammation such as IL-6, the principal regulator of the acute-phase response [7]. IL-10 is an anti-inflammatory cytokine that strongly inhibits the synthesis of pro-inflammatory cytokines at the transcriptional level by controlling the degradation of the inhibitory protein of NF-κB, IκB, and thereby the nuclear translocation of NF-κB [8]. IL-10 has a central role in the control and termination of systemic inflammation. Although IL-10 is thought to have a protective role in the early postoperative period, the maintenance of normal postoperative organ function is likely to depend on an adequate balance between the production of pro-inflammatory and anti-inflammatory cytokines [9]. It has been suggested that the overproduction of IL-10 after severe injury might be associated with a hyporesponsiveness to lipopolysaccharide (LPS) that carries a higher risk for infections [10].

The *ex vivo *production of cytokines by whole blood is a widely accepted method of evaluating the reactivity of immunoreactive and inflammatory cells and their potential for inflammatory responses [11]. In this study, we tested the hypothesis that neonatal cardiac surgery would influence the *ex vivo *production of cytokines.

## Materials and methods

### Patients

After approval by the Human Ethical Committee of the Aachen University Hospital as well as written consent from the parents, 17 consecutive newborn infants aged 2 to 13 days (median 8 days) were included in this study. To ensure homogeneity of the patient group, the inclusion criterion was a simple transposition of the great arteries, suitable for an arterial switch operation. All patients received prostaglandin E_1 _infusion (0.05 μg kg^-1 ^min^-1^) before the operation, to maintain patency of the ductus arteriosus. Preoperative cardiac catheterization for balloon atrioseptostomy and angiography was performed in 13 patients.

### Anesthesia, operative management and postoperative care

Conventional general anesthesia was conducted with diazepam, fentanyl sulfate and pancuronium bromide. Perioperative antibiotic prophylaxis consisted of cefotiam hydrochloride (100 mg kg^-1 ^body weight). Dexamethasone (10 mg m^-2 ^body surface area) was administered immediately before sternotomy.

The standardized neonatal cardiopulmonary bypass (CPB) protocol included a roller pump, a disposable membrane oxygenator and an arterial filter. All patients were operated on under deep hypothermic CPB, as described previously [5]. Epinephrine (adrenaline), dopamine and sodium nitroprusside were administered systemically for weaning the patients from CPB.

Standardized postoperative care was provided. Monitoring included continuous registration of hemodynamic variables, diuresis and blood gases. Inotropic support consisted in all cases of dopamine (5 μg kg^-1 ^min^-1^) and, if necessary, epinephrine (0.05 to 0.2 μg kg^-1 ^min^-1^) or dobutamine (5 to 7.5 μg kg^-1 ^min^-1^) and vasodilatory treatment of sodium nitroprusside (0.5 to 2 μg kg^-1 ^min^-1^). Diuretics (furosemide, single dosage of 0.1 to 1 mg kg^-1^) and volume substitution, which consisted of fresh-frozen plasma or human albumin 5%, were administered depending on the hemodynamic variables. Postoperative clinical endpoint variables were mean arterial blood pressure, mean central venous pressure, need for inotropic support, oxygenation index expressed as the ratio of partial arterial oxygen tension to fraction of inspired oxygen, minimal diuresis, maximal serum creatinine and maximal serum glutamate oxaloacetate transaminase values during the first 72 hours after the operation, and duration of inotropic and ventilatory support.

### Blood elements

Leukocyte counts were determined by a Cell-Dyn 3700 (Abbott GmbH & Co. KG, Wiesbaden, Germany).

### C-reactive protein

C-reactive protein was determined by laser nephelometry. The detection limit of this method is 5 mg dl^-1^.

### *Ex vivo *stimulation

Whole blood culture was performed as described previously [12]. Blood (1 ml) was withdrawn under sterile conditions from a peripheral vein and was taken in endotoxin-free tubes (Endo tube ET; Chromogenix, Haemochrom Diagnostica GmbH, Essen, Germany) before the operation (median 5 days), as well as 5 and 10 days after operation. The timing of blood samples was dictated by the fact that *ex vivo *production of TNF-α was reported to be decreased up to the sixth postoperative day in adults undergoing cardiac surgery [13]. Blood was mixed in a 1:10 ratio with RPMI 1640 medium containing L-glutamine and 25 mM Hepes medium (Bio Whittaker Europe, Verviers, Belgium). Cell cultures were stimulated with LPS (LPS for cell culture, *Escherichia coli*, lot 026.B6:L2654; Sigma, St Louis, MO, USA) at a final concentration of 1 ng ml^-1^. In control samples, the LPS volume was replaced with cell culture medium. Because it has been shown that *ex vivo *cytokine production reaches its plateau mainly between 12 and 24 hours after stimulation [14], cell cultures were incubated for 16 hours in a humidified incubator at 37°C in an atmosphere consisting of a mixture of 5% CO_2 _and 95% air (Heraeus HBB 2472b; Heraeus Instruments GmbH, Hanau, Germany); the supernatant was then separated after centrifugation (2,500 r.p.m. for 3 min) and frozen at -70°C until assay.

### Cytokine determination

TNF-α, IL-6 and IL-10 were determined with an immunocytometric assay (Biosource International, Camarillo, CA, USA), in accordance with the manufacturer's recommendations for cell culture supernatant. It is a solid-phase, enzyme-amplified sensitivity immunoassay performed on microtiter plates based on the oligoclonal system in which several monoclonal antibodies directed against distinct epitopes of cytokines are used, permitting a high sensitivity of the assay. The minimal detectable concentrations are 3 pg ml^-1 ^for TNF-α, 2 pg ml^-1 ^for IL-6, and 1 pg ml^-1 ^for IL-10. The ranges covered by the standard curve are 0 to 1,700 pg ml^-1 ^for TNF-α, 0 to 2,100 pg ml^-1 ^for IL-6, and 0 to 1,750 pg ml^-1 ^for IL-10. Samples were diluted accordingly.

### Statistical analysis

Results are expressed as means ± SEM. The data were analyzed with the nonparametric paired Wilcoxon rank test. The Spearman rank correlation coefficient was assessed for correlation of independent parameters. *P *< 0.05 was considered significant.

## Results

### Clinical results

Operative data and clinical results are summarized in Table 1. Seven of the 17 newborn infants showed early postoperative complications that are summarized in Table 2. Six of the seven patients with complications had a capillary leak syndrome as previously described by our group [15]. One patient developed pneumonia. There was one postoperative death 29 days after operation in a patient having developed thrombosis of the right and of the left persistent superior caval veins.

### Leukocyte count

There was no statistical difference between the counts of leukocytes, granulocytes and monocytes measured before the operation, and 5 and 10 days after it (Table 3). Leukocyte counts were not different in patients with or without complications.

### C-reactive protein

C-reactive protein (CRP) increased in all patients from 7.94 ± 1.27 mg dl^-1 ^before the operation to 15.7 ± 3.7 mg dl^-1 ^5 days after it. At that time point, CRP values were higher in patients with complications than in those without (23.8 ± 5.5 versus 10.2 ± 4.3 mg dl^-1^, *P *= 0.001). The patient with pneumonia had a CRP value of 8 mg dl^-1 ^before the operation and 9 mg dl^-1 ^5 days after the operation, increasing to 50 mg dl^-1 ^12 hours later. CRP values were still elevated in all patients 10 days after the operation (16.6 ± 4.4 mg dl^-1^), and at that time there was no difference between patients with and without complications. The patient with pneumonia had a CRP value of 8 mg dl^-1 ^at that time.

### *Ex vivo *production of cytokines after LPS stimulation before and after operation

At all time points investigated in this study there was a significant production of TNF-α, IL-6 and IL-10 after stimulation by LPS in comparison with the control sample.

Concentrations of TNF-α and IL-6 in the cell culture supernatant were significantly decreased on day 5, in comparison with preoperative levels (*P *< 0.001 and *P *< 0.002, respectively).

**Figure 1:**
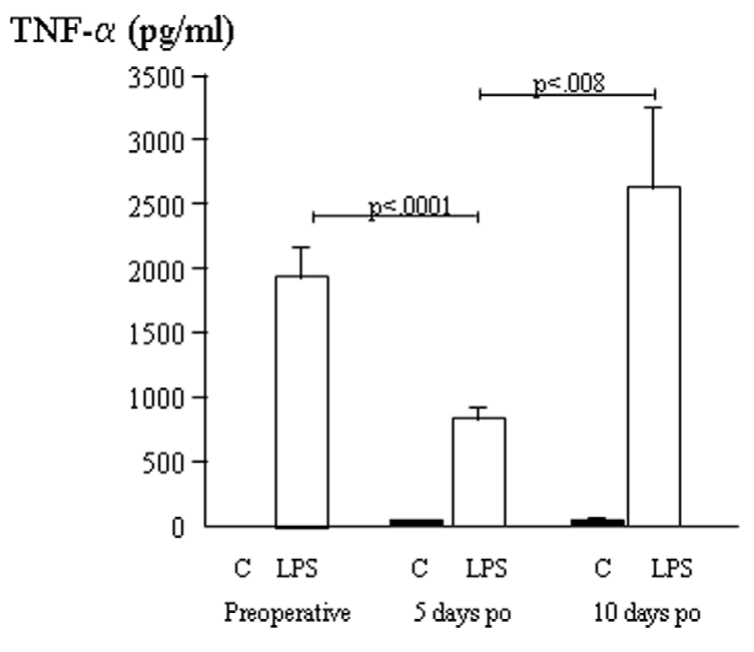
*Ex vivo *production of tumor necrosis factor-α. Preoperative and postoperative (po) tumor necrosis factor-α (TNF-α) levels in whole blood culture supernatant. Values are expressed as means and SEM (error bars). TNF-α production was significantly increased after stimulation with lipopolysaccharide (LPS; white), in comparison with the unstimulated control (C; black) at all time points. In comparison with preoperative levels, TNF-α production after stimulation with LPS significantly decreased 5 days after operation (*P *< 0.001) but again reached preoperative levels 10 days after operation.

**Figure 2:**
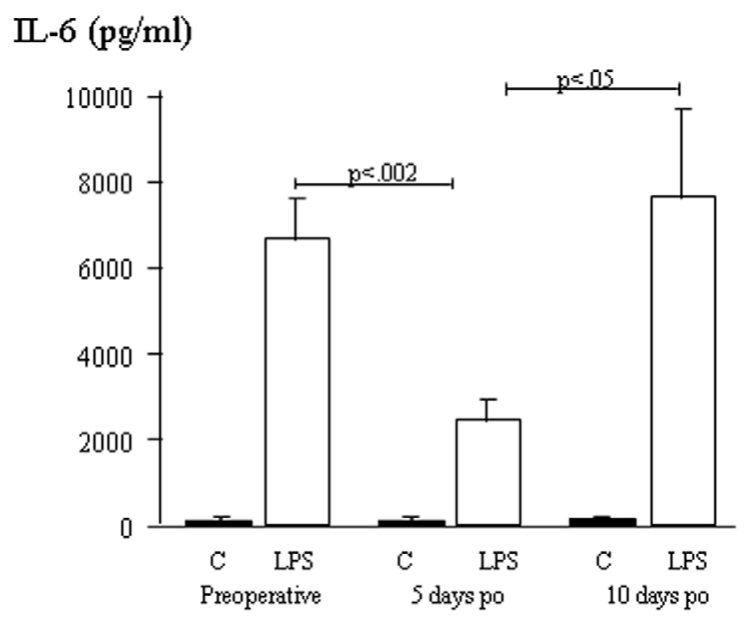
*Ex vivo *production of interleukin-6. Preoperative and postoperative (po) interleukin (IL)-6 levels in whole blood culture supernatant. Values are expressed as means and SEM (error bars). IL-6 production was significantly increased after stimulation with lipopolysaccharide (LPS; white), in comparison with the unstimulated control (C; black) at all time points. In comparison with preoperative levels, IL-6 production after stimulation with LPS significantly decreased 5 days after operation (*P *< 0.002) but again reached preoperative levels 10 days after operation.

**Figure 3:**
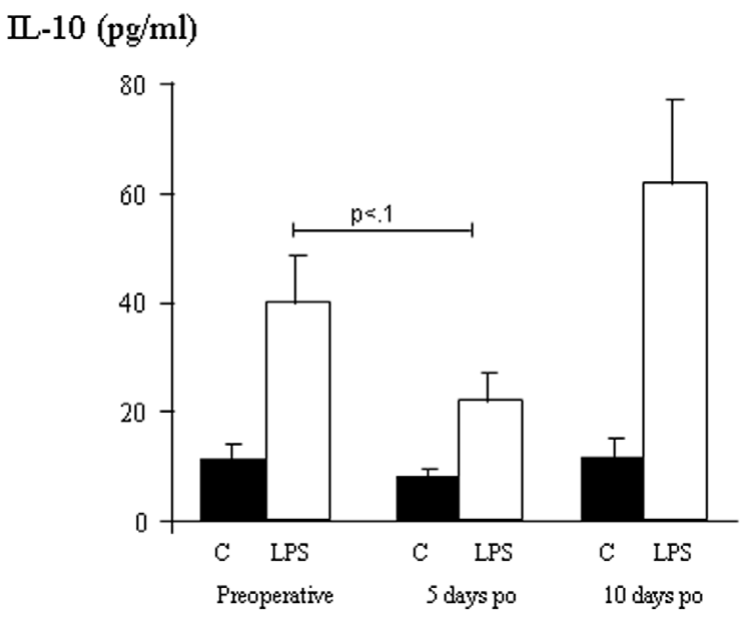
*Ex vivo *production of interleukin-10. Preoperative and postoperative (po) IL-10 levels in whole blood culture supernatant. Values are expressed as means and SEM (error bars). IL-10 production was significantly increased after stimulation with lipopolysaccharide (LPS; white), in comparison with the control (C; black) at all time points. In comparison with preoperative levels, IL-10 production after stimulation with LPS tended to decrease 5 days after operation but again reached preoperative levels 10 days after operation.

Postoperative IL-10 concentrations on day 5 were also reduced compared with the preoperative value, although not significantly (*P *< 0.1). On the 10th day after the operation, concentrations of TNF-α, IL-6 and IL-10 had returned to their preoperative levels (Figs 1, 2, 3).

### Correlation between *ex vivo *production of cytokines and outcome

**Figure 4:**
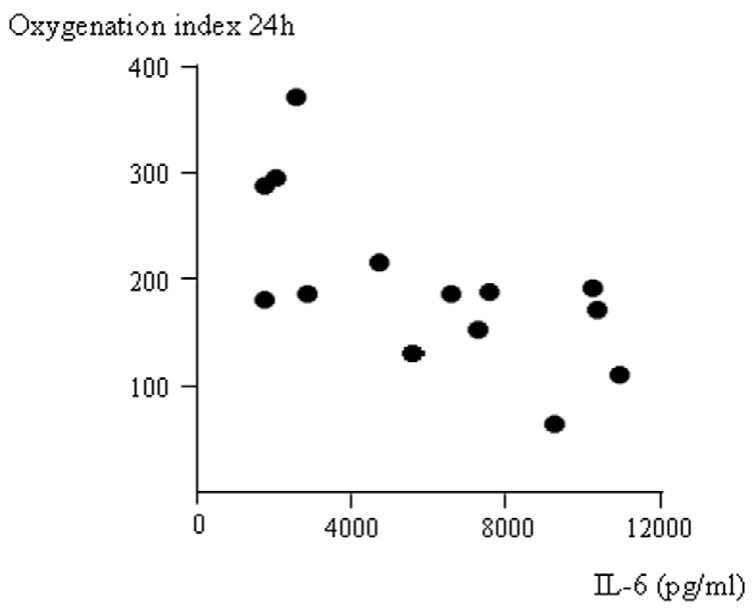
Relationship between preoperative production of interleukin-6 (IL-6) and postoperative pulmonary dysfunction. Plot showing the correlation between preoperative IL-6 production after stimulation with lipopolysaccharide (LPS) and the oxygenation index 24 hours after operation (*n *= 14). Spearman correlation coefficient -0.62; *P *< 0.02.

In all patients preoperative IL-6 production was inversely correlated with the oxygenation index, as measured 4 and 24 hours after the operation (Spearman correlation coefficient: -0.62; *P *< 0.02). Figure 4 shows the relationship between preoperative *ex vivo *IL-6 production and the oxygenation index, as measured 24 hours after the operation. There was no correlation between the *ex vivo *production of TNF-α and IL-10 and postoperative morbidity, respectively. In particular, the only patient with pneumonia (patient 2 in Table 2) showed *ex vivo *cytokine production that was in the same range as for all other patients.

## Discussion

In previous studies we have shown that neonatal cardiac surgery induces a systemic inflammatory reaction with complement activation, leukocyte stimulation and cytokine synthesis that is associated with postoperative complications such as the capillary leak syndrome and myocardial dysfunction [2,5,15]. In this study we confirm the association between systemic inflammation and postoperative morbidity. Although it has been suggested that, in the setting of cardiac surgery, parenchymatous cells such as cardiomyocytes contribute to the systemic inflammatory reaction by producing cytokines, circulating blood cells, in particular leukocytes, are considered the major source of inflammatory mediators [16,17]. This is supported by previous studies that report a clear association between uncontrolled leukocyte activation and early postoperative morbidity after cardiac surgery in newborn infants and in children [5,15].

The systemic inflammatory reaction induced by cardiac surgery is normally controlled by a natural anti-inflammatory response. Indeed, levels of IL-10 are already increased at the end of the operation and remain substantially elevated for at least 48 hours after the operation [18].

Although the anti-inflammatory response to cardiac surgery is thought to be beneficial with regard to early postoperative organ protection [17], it remains unclear whether it could impair leukocyte reactivity and thereby decrease resistance against infections.

In this study, the reactivity of circulating cells after neonatal cardiac surgery was evaluated by the *ex vivo *production of pro-inflammatory and anti-inflammatory cytokines after a standardized inflammatory stimulus in a homogenous patient group.

A previous study in older children who had undergone cardiac surgery for various cardiac defects showed decreased *ex vivo *cytokine production on the morning of the first postoperative day. However, later time points, to document the normalization of cytokine production, were not investigated [19]. One main result of our study is that neonatal cardiac surgery is associated with a transiently decreased *ex vivo *production of the pro-inflammatory cytokines TNF-α and IL-6, and that this is not related to a decrease in leukocyte count. This indicates impaired reactivity of inflammatory cells. In adults this phenomenon has been reported after cardiac surgery [13], severe injury and sepsis, and defined as hyporesponsiveness to LPS [20,21]. In adults who have undergone cardiac surgery, *ex vivo *TNF-α production and TNF-α mRNA in whole blood were still lower at the end of the study period, which was 6 days after surgery [13]. We also investigated the *ex vivo *production of cytokines at a later time point and show a return of TNF-α production to preoperative values 10 days after cardiac surgery. The reason for the transient impairment of leukocyte reactivity in our series could be ascribed to the exhaustion of circulating inflammatory cells due to the massive inflammatory stress due to cardiac surgery and also to the perioperative treatment applied. With this regard, drugs administered before, during and after the operation could have influenced hyporesponsiveness to LPS. Indeed, prostaglandin E_1 _has been shown to reduce the *ex vivo *production of TNF-α and IL-1β by adult monocytes [22]. However, in our patients who were all treated with prostaglandin-E_1 _infusion before the operation, preoperative levels of cytokines measured in the supernatant of the whole blood culture were similar to those after stimulation of cord blood in healthy newborn infants [12]. This suggests a minor effect of prostaglandin-E_1 _on the *ex vivo *production of cytokines in our study. In adults, anesthesia and heparin were shown not to influence the *ex vivo *production of TNF-α [13].

The course of *ex vivo *IL-10 production after cardiac surgery has so far not been followed for more than 6 hours after CPB. In a previous study, IL-10 production reached its lowest point 2 hours after cardiac surgery in adult patients and returned to preoperative values 6 hours later [10]. Our results, in contrast, show that in newborn infants the *ex vivo *production of IL-10 was decreased 5 days after the operation, even though not significantly in comparison with preoperative values. This reduction could be the result of negative feedback by IL-10, which inhibits not only pro-inflammatory cytokines but also its own production.

The exact mechanisms leading to hyporesponsiveness to LPS in newborn infants reported here are not yet clear. However, the anti-inflammatory cytokines IL-10 and tissue growth factor-β are thought to be important in its regulation [23]. In a clinical study, adult patients with sepsis or severe trauma showed a reduced expression of the active form of NF-κB [24]. In those who did not survive, the IL-10 plasma levels were inversely correlated to the ratio between the active and inhibitory forms of NF-κB, supporting the view that IL-10 might participate in the induction of LPS hyporesponsiveness by inhibiting cytokine synthesis at or upstream of the transcriptional level.

Newborn infants who have undergone cardiac surgery have been reported to show a higher natural production of IL-10 than older children [25]. For the reasons cited above, this natural anti-inflammatory cytokine imbalance could well have contributed to the hyporesponsiveness to LPS observed in our series. Furthermore, as reported by others in older children operated on with CPB [19], perioperative treatment with dexamethasone could also have contributed to hyporesponsiveness to LPS by inhibiting the activation of NF-κB and thereby the production of pro-inflammatory cytokines [26], as well as by stimulating the production of IL-10 [27,28].

Although a clear association has been demonstrated between hyporesponsiveness to LPS and poor clinical outcome in sepsis [24], we were not able to confirm such an association in our series. One reason for this might be that, in the small group of patients investigated, the overall rate of complications related to inflammation or infection was low.

In contrast, we observed a clear association between the preoperative *ex vivo *production of IL-6 and postoperative respiratory morbidity. This suggests that a higher preoperative potential for *ex vivo *production of IL-6 is a risk factor for inflammation-related postoperative complications in newborn infants.

## Conclusion

Our results show for the first time that cardiac surgery in newborn infants is associated with a transient but significant decrease in the *ex vivo *production of the pro-inflammatory cytokines TNF-α and IL-6 together with a less pronounced decrease in IL-10 production. This suggests a postoperative anti-inflammatory shift of the cytokine balance in this age group 5 days after cardiac surgery. A higher preoperative *ex vivo *production of IL-6 might indicate a higher risk for postoperative pulmonary dysfunction. Further studies will address the question of whether preoperative *ex vivo *production of IL-6 would be a suitable predictor of postoperative complications in newborn infants with congenital cardiac defects.

## Key messages

• 	Cardiac surgery in newborn infants decreases the reactivity of blood cells to LPS.

• 	Cardiac surgery in newborn infants might lead to an anti-inflammatory shift of the cytokine balance.

• 	In this series, postoperative complications related to decreased blood cell reactivity were not observed.

• 	The higher the *ex vivo *production of IL-6 before the operation, the worse the postoperative lung function.

• 	Testing the *ex vivo *production of IL-6 in newborn infants might help to predict postoperative pulmonary dysfunction.

## Abbreviations

CPB = cardiopulmonary bypass; CRP = C-reactive protein; IL = interleukin; LPS = lipopolysaccharide; NF-κB = nuclear factor κB; TNF-α = tumor necrosis factor-α.

## Competing interests

The author(s) declare that they have no competing interests.

## Authors' contributions

KS performed whole blood cultures, ELISAs, acquisition and statistical analysis of the data, and redaction of the manuscript. SK performed ELISAs, data acquisition and analysis. JFV-J coordinated sample withdrawal and revised the manuscript. GvB drafted the manuscript and revised it critically. JD supervised the blood cultures and ELISAs, study design, data analysis and interpretation. M-CS was responsible for study conception and design, data analysis and interpretation and manuscript preparation and final revision. All authors read and approved the final manuscript.

## Figures and Tables

**Table 1:** Clinical and operative data

Variable	Value
Age at operation (days)	8 (2–13)
Duration of cardiopulmonary bypass (min)	58 (53–63)
Duration of aortic cross-clamping (min)	62 (54–78)
Mean blood pressure (mmHg)	
4 h after operation	65 (48–80)
24 h after operation	53 (47–68)
Diuresis (ml kg^-1 ^h^-1^)	
4 h after operation	7.8 (1.6–17.5)
24 h after operation	7.1 (1–8)
Oxygenation index PaO_2_/FiO_2 _(mmHg)	
4 h after operation	176.7 (69–283)
24 h after operation	195.5 (63–370)
Aspartate aminotransferase concentration (IU L^-1^)	
4 h after operation	32 (13–66)
24 h after operation	33 (7–162)
Epinephrine dosage (μg kg^-1 ^min^-1^)	
4 h after operation	0.16 (0.02–0.36)
24 h after operation	0.12 (0.02–0.41)

Values are presented as number (*n*) and range. FiO_2_, fraction of inspired oxygen; PaO_2_, partial arterial oxygen tension.

**Table 2:** Postoperative complications

Patient	Complications	Time after operation	Outcome
1	Cardiac arrest after blood transfusion	4 h	Survived
2	Capillary leak syndrome^a^	24 h	Survived
	Pneumonia^b^	5 d	
3	Capillary leak syndrome	24 h	Survived
4	Capillary leak syndrome	24 h	Survived
5	Capillary leak syndrome	24 h	Survived
6	Capillary leak syndrome	24 h	Survived
7	Capillary leak syndrome	24 h	Died
	Thrombosis of the right and left persistent superior caval veins	10 d	

^a^Capillary leak syndrome was diagnosed in accordance with our definition [15]. ^b^Diagnosis of pneumonia was made on the basis of respiratory insufficiency, a pathological chest X-ray and a secondary increase in C-reactive protein.

**Table 3:** Preoperative and postoperative leukocyte, granulocyte, monocyte and lymphocyte counts

Cell type	Cell count		
	
	Before operation	5 d after operation	10 d after operation
Leukocytes (Giga l^-1^)	12.8 ± 1.2	10.1 ± 0.8	11.9 ± 1.3
Granulocytes (%)	53.2 ± 4.2	55.9 ± 3.1	45.2 ± 4.5
Monocytes (%)	3.7 ± 1.5	3 ± 0.6	9.1 ± 1.6
Lymphocytes (%)	37.4 ± 5.9	33.4 ± 4	37.5 ± 3.8

Values are presented as means ± SEM.
